# Editorial Notes

**Published:** 1877-12

**Authors:** 


### Editorial.

---

THE NUMEROUS medical publications in every portion of the civilized world, afford the means of commnnicating and receiving items tending to advancement in, and perfection of, the science of medicine. Were all the newly-discovered facts thrown into this vast chain of media, certainly more rapid strides toward perfection in the healing art would be the result. Unfortunately, however, the most devoted, unselfish votaries of science content themselves with making such discoveries practically useful, only to those with whom they are immediately associated.

Notwithstanding this failure, much of the material calculated to promote advancement in medicine, finds its way into these channels, and real progress is certainly being had. There is no lack of compilers to bring the scattered fund from periodicals and other sources, into the more permanent repository of text-books; but the faithfulness of some is rather questionable. It is easier to dress up in a fascinating style, old and exploded ideas, gathered from standard works, than to collect and arrange correct principles from less tangible sources. Hence, some of the new works show no very decided advancement on the subjects of which they treat. Moreover, the books are made to sell—at any rate, this is one of the objects of their manufacture—and decided innovations upon long-taught and cherished principles are likely to interfere with the desired pecuniary returns.

For a few months past, our engagements with other matters have prevented reviews and notices of new publications to the extent desirable. Quite a number of excellent monographs have been received during the year, many of which are worthy of extended notice. This form of communication with the medical public, on special subjects, is interesting and profitable.

Some of the valuable books sent us from the publishing houses of New York, Philadelphia and other places, have received only a mere notice, but so soon as opportunity is afforded, we propose yet to speak more at length of their merits. We shall not hesitate to mention, also, what of defects we may believe they possess. As a journalist, we cannot consent to laud error and be the means of encouraging, unjustly, the sale of books that will not, in our opinion, benefit the profession and the public.

